# Enhancing Stroke Prevention in Transcatheter Aortic Valve Replacement: The Role of F2, a Novel Neuroprotection Device

**DOI:** 10.1007/s13239-025-00806-w

**Published:** 2025-10-01

**Authors:** Mahsa Ghovvati, Taichiro Imahori, Nina Fukui, Kenichi Sakuta, Yoshiki Hanaoka, Lea Guo, Amir M. Molaie, Aryan M. Gajjar, Satoshi Tateshima, Gary Duckwiler, Naoki Kaneko

**Affiliations:** 1 Department of Radiological Sciences, David Geffen School of Medicine at UCLA, Los Angeles, California USA; 2 Department of Neurosurgery, Kitaharima Medical Center, Hyogo, Japan; 3 Department of Neurology, Jikei University School of Medicine, Tokyo, Japan; 4 Department of Neurosurgery, Shinshu University School of Medicine, Matsumoto, Japan; 5 Department of Neurology, David Geffen School of Medicine at UCLA, Los Angeles, California USA

**Keywords:** Transcatheter aortic valve replacement (TAVR), Cerebral embolic protection (CEP), Stroke prevention, Embolic debris, F2 filter

## Abstract

**Purpose:**

Transcatheter aortic valve replacement (TAVR) is an established treatment for severe aortic stenosis; however, it carries the risk of periprocedural strokes. Current cerebral embolic protection (CEP) devices, such as the Sentinel, provide partial protection but are limited by inadequate anatomical coverage and inability to capture smaller emboli effectively. This study aimed to evaluate the effectiveness of a novel CEP device, the F2 filter with a 28 μm pore size and full cervical vessel coverage, in preventing emboli from entering the cerebral circulation.

**Methods:**

The Sentinel and F2 filter were evaluated for the ability to prevent embolic particles of various sizes (45-300 µm) from entering cerebral arteries using two in vitro flow models, incorporating standard and tortuous aortic anatomies. Additionally flow rates were also measured to confirm that normal perfusion was maintained while the devices were in place.

**Results:**

The F2 filter maintained normal cerebral arterial flow and significantly reduced the number of particles across all sizes compared to the Sentinel and control groups. This reduction was observed in all four cerebral branches and across both standard and tortuous aorta models.

**Conclusions:**

The F2 filter showed superior neuroprotective effectiveness to prevent embolic debris from entering the cerebral circulation in the in vitro models. By offering comprehensive coverage to all cervical arteries and with a smaller mesh size, this filter has the potential to improve cerebral protection during TAVR.

**Supplementary Information:**

The online version contains supplementary material available at 10.1007/s13239-025-00806-w.

## Introduction

Transcatheter aortic valve replacement (TAVR) is an established treatment for severe, symptomatic aortic stenosis [1]. However, major stroke occurs in 3% to 10% of patients undergoing TAVR [2–5]. In addition, silent brain infarcts (SBIs) detected by diffusion-weighted magnetic resonance imaging (DW-MRI) affects 68 to100% of patients after TAVR [6–10]. While the clinical impact of SBIs in TAVR patients is not fully understood, they have been linked to cognitive decline and dementia [11].

Cerebral embolic protection (CEP) devices are designed to minimize the risk of stroke during TAVR by capturing or deflecting embolic debris [12]. Several CEP devices have been developed, which vary in their access site, sheath size, mesh pore diameter, and extent of cerebral arteries protection [13–18]. The ideal CEP device should provide full cerebral protection, be easy and safe to use and position, and remain stable throughout the procedure [19, 20]. The only FDA-approved CEP device, the Sentinel (Boston Scientific, Marlborough, MA, USA), has inadequate anatomical coverage, leaving the left vertebral artery unprotected. Its large pore size also limits its ability to capture smaller emboli. This lack of full coverage with its large pore size may explain why some clinical studies have shown that the Sentinel device has not demonstrated a significant reduction in the volume of new cerebral ischemic lesions [10, 12].

Given the limitations of the Sentinel which include inadequate anatomical coverage and limited ability to capture smaller debris, there is a need for new devices that offer more comprehensive cerebral protection during TAVR. The F2 filter (EnCompass Technologies Inc., CA, USA) is a self-expanding nitinol stent with a smaller-pore filter membrane (28 μm), designed to provide full coverage of all four cerebral arteries and to capture smaller emboli.

In this study, CEP devices were evaluated for their ability to maintain cerebral blood flow and prevent emboli of various sizes from entering the cerebral circulation using two realistic aorta models.

## Methods

### F2 Filter Device Deployment and Procedural Sequence

**Fig. 1:**
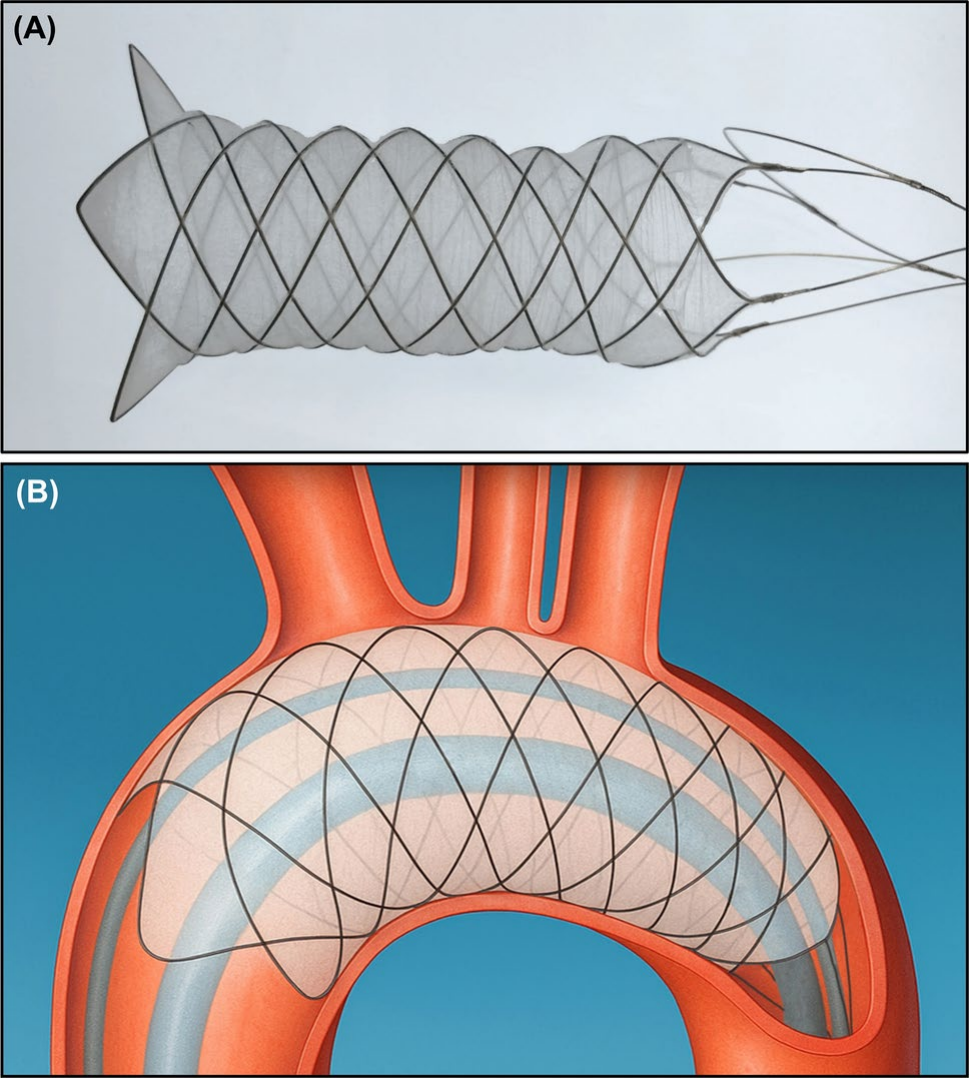
**a** A representative image of EnCompass F2 filter, and **b** Schematic of deployment and use of EnCompass F2 filter. The F2 filter device is delivered from the femoral artery and deployed in the aortic arch by unsheathing to cover the three great vessels. After full expansion and wall apposition, the TAVR system passes through the open central lumen of the F2 filter. The filter remains in place throughout the procedure and protects the brain by diverting procedure generated debris away from all cervical arteries

The F2 filter is a temporary self-expanding nitinol stent with a filter membrane made of electrospun polyurethane coated with hydrophilic heparin. The membrane, with a pore size of 28 μm, diverts emboli away from the cerebral circulation. The device is deployed in the aortic arch via a transfemoral approach, utilizing a simple unsheathing technique to position it over all the three great vessels from the aorta. (Figures 1a and 1b).

### Development of the In Vitro Flow Model

**Fig. 2:**
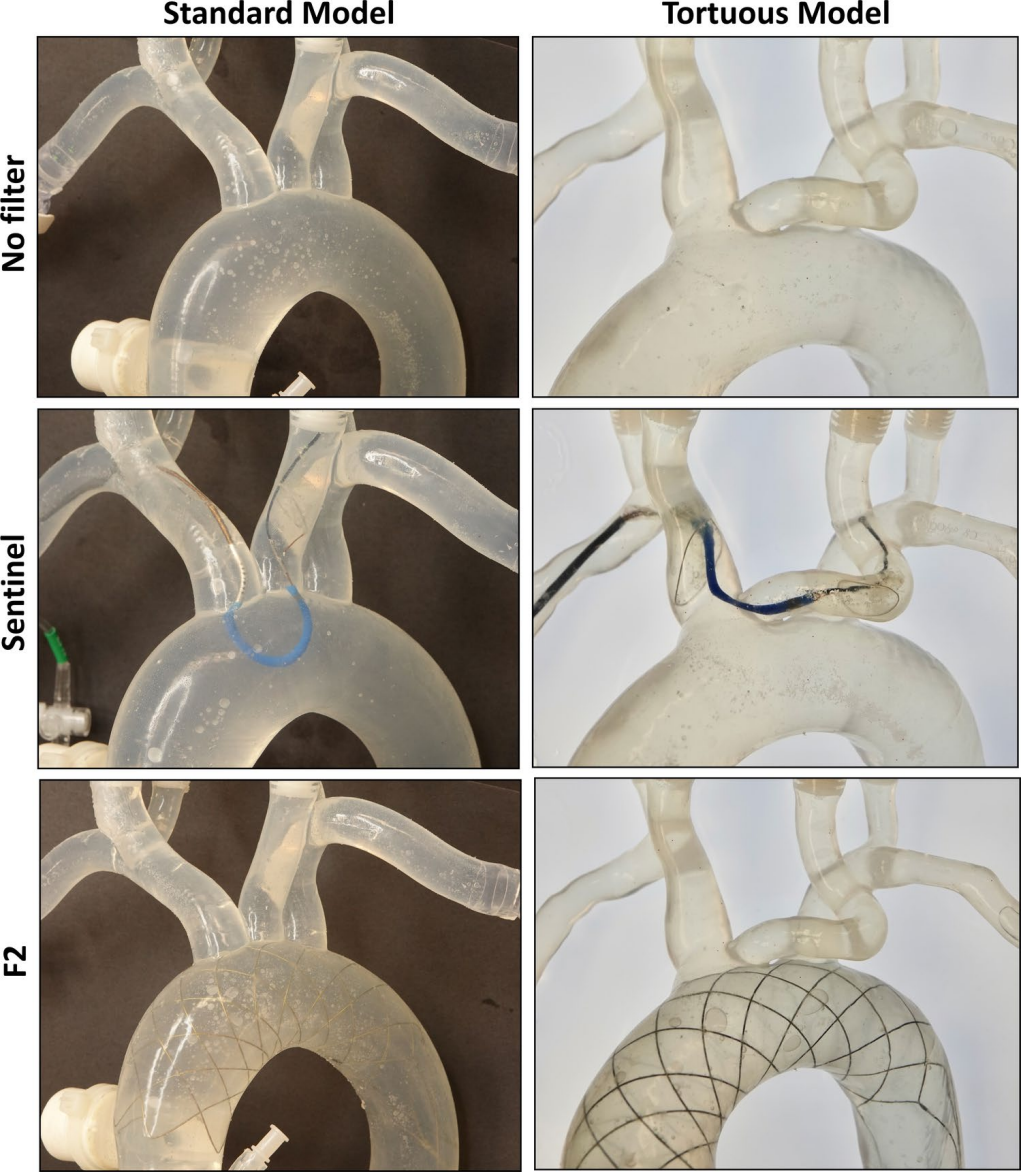
Images of the Sentinel and F2 filters implanted in the aortic arch silicone models (standard and tortuous models)

Two silicone aortic arch models, one standard and one tortuous, were obtained from Mentice Inc (Chicago, Illinois, USA). Each model included the human aortic arch and major great vessels branches, including the bilateral common carotid and vertebral arteries (Figure 2). To replicate physiological flow, the models were connected to a peristaltic pump (MasterFlex®, Model: 77601-10) and perfused with a phosphate-buffered saline (PBS) solution containing 0.03% (w/v) xanthan gum (Xanthomonas campestris; Sigma Aldrich/MilliporeSigma) and 0.05% (v/v) Tween 20 (Fisher). This working fluid was adjusted to a dynamic viscosity of 3.95 cP at 37 °C and a shear rate of 10 s⁻^1^, closely approximating whole blood viscosity under low-shear conditions typical of large-vessel flow. A digital thermostatic water bath maintained the system temperature at 37 °C, and a mesh filter was placed in the bath to prevent particle recirculation. The total circulating flow rate was 5 L/min. Each branch and the descending aorta discharged to atmosphere into separate collection reservoirs. Flow distribution to individual branches was monitored using a tubing flow meter (Transonic Systems Inc., Model: TS410 & ME5PXL, NY, USA) and set by adjusting outlet height and in line resistors as follows: 80 mL/min for the right and left vertebral arteries (RVA and LVA), 400 mL/min for the right and left common carotid arteries (RCCA and LCCA), and 250 mL/min for the right and left subclavian arteries (RSCA and LSCA).

### Deployment of CEP Devices and Flow Volume Analysis

The CEP devices, F2 filter and commercially available Sentinel, were used with their respective delivery systems according to the manufacturers’ instructions. After each particle injection, the device was removed and rinsed thoroughly to eliminate retained particles. If the device remained structurally intact, it was redeployed in the same anatomical location up to 3 times. If any damage or deformation was noted, it was replaced with a new unit. The volumetric flow rate (mL/min) in each cerebral artery was measured for the control (no protection) and for each CEP condition (F2 and Sentinel). Before every experiment, the system was operated and branch flow in each artery was measured for consistency.

### Particle Injection Procedure and Quantitative Analysis

To evaluate the efficacy of the CEP devices in preventing particles from entering the cerebral circulation, a 10 mL dose of polyethylene microspheres (Cospheric LLC, CA, USA; particle density: 1.00 g/cc) suspended in Xanthan/Tween^®^20 solution was injected into the base of the ascending aorta. The microsphere suspension contained 0.75 mg/mL of small particles (45–53 µm), 3.37 mg/mL of medium particles (106–125 µm), and 18 mg/mL of large particles (250–300 µm). The concentrations were chosen to over‑weight large particles by mass while keeping small particles numerically predominant, reflecting clinical debris patterns in which small particles are most numerous but larger fragments contribute most of the cumulative area/mass. Using the manufacturer’s spheres‑per‑gram table for polyethylene beads, the mixture yields approximately 12,175 small, 4,177 medium, and 1,653 large particles per mL (≈68%/23%/9% by count) but ≈3.4%/15.2%/81.4% by mass. The particles were injected for 20 seconds, and the pump was allowed to run for an additional 10 seconds. The solution collected from RVA, RCCA, LCCA, and LVA was then passed through strainers (PluriSelect, CA, USA) with pore sizes of 200 µm, 85 µm, and 30 µm, in descending order, to separate the three particle sizes. Particle injection experiments were performed nine times for each group.

To determine the number of the collected particles, a Multisizer Coulter Counter (Multisizer 4e, Beckman Coulter^®^) was used. In this method, particles pass through a small aperture within the counter, altering the electrical resistance of the fluid between electrodes and generating a pulse. Counting and analyzing these pulses provide information about the number and size distribution of the particles [21]. For each sample, 20 mL of Isoton II solution (Beckman Coulter^®^) was added to the particles obtained from each strainer in a cuvette chamber. Appropriate aperture sizes were selected to accommodate the different particle size ranges. To validate the accuracy of the counting system, control runs were performed by preparing reference suspensions with known particle concentrations based on the manufacturer’s bead specifications.

Across the nine replicate experiments per group, the number of particles collected from each size range at the branch outlets showed low variability, demonstrating the consistency and reproducibility of the collection and measurement process. Moreover, to confirm that the particle counts represented polyethylene microspheres rather than artifacts, we ran Isoton blanks between samples, which yielded background counts < 0.05% of sample values, indicating negligible contribution from air bubbles.

### Protection Efficacy Definition and Statistical Analysis

Comparative analyses were performed to evaluate the effectiveness of the CEP devices versus controls (without CEP devices) for particles of varying sizes (45–53 µm, 106–125 µm, and 250–300 µm) as they entered the major cerebral arteries.

The protection efficacy $\left( \eta \right)$. of the CEP devices was defined with the following equation:

$$\eta = 100 \times \left( {1 - \frac{Average\,number\,of\,particles\,in\,filtered\,cerebral\,arteries\,with\,CEP\,device}{{Average\,number\,of\,particles\,in\,cerebral\,arteries\,without\,protection}}} \right)$$

A p-value of ≤ 0.05 was considered statistically significant. Data were analyzed using either one-way or two-way ANOVA techniques, depending on the variables present in the dataset, utilizing GraphPad Prism 8 software.

## Results

### Effect of CEP Devices on Cerebral Arterial Flow Rates

**Fig. 3:**
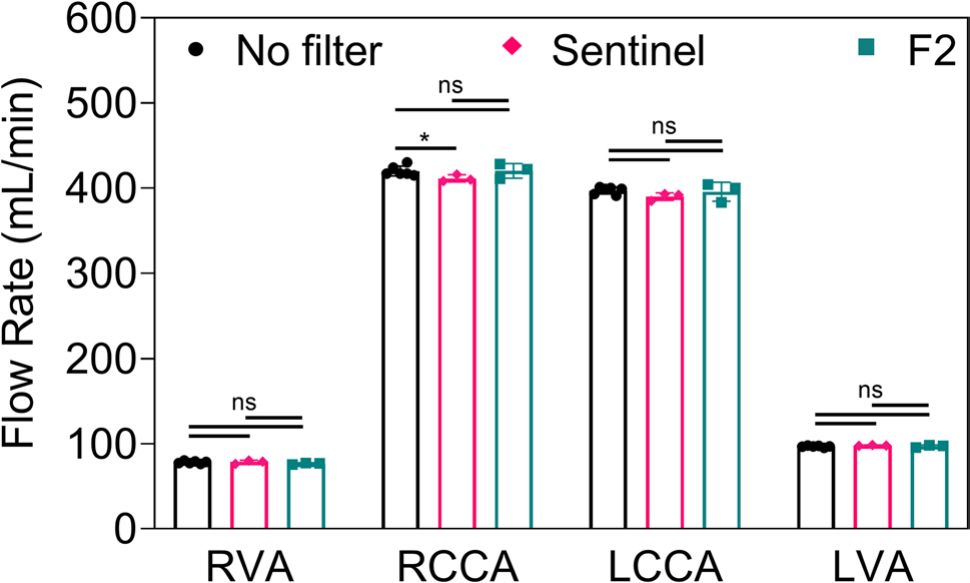
Volumetric flow rate (mL/min) of Xanthan/Tween^®^20 solution in each of cerebral arteries with and without CEP devices (*i.e.* Sentinel and F2 filters) (n = 6 for control group (without protection), and n = 3 for each of CEP devices); asterisk mark significance level of p ≤ 0.05 (*). ***Abbreviations:*** *CEP* Cerebral embolic protection, *RVA* Right vertebral artery, *RCCA* Right common carotid artery, *LCCA* Left common carotid artery, *LVA* Left vertebral artery

To assess whether the devices, particularly the F2 filter with its smaller pore size, would reduce cerebral flow, the volumetric rate (mL/min) were measured in each cerebral artery, with and without the Sentinel and F2 devices in place (Figure 3). There was no discernible alteration in the flow rate within all cerebral arteries with the F2 filter compared to the unprotected control groups. Conversely, the use of the Sentinel device led to a slight but statistically significant decrease in flow rate within the RCCA compared to the unprotected control groups (p ≤ 0.05), while no significant differences were observed in the other arteries.

### Efficacy Comparison of Embolic Protection Devices

**Fig. 4:**
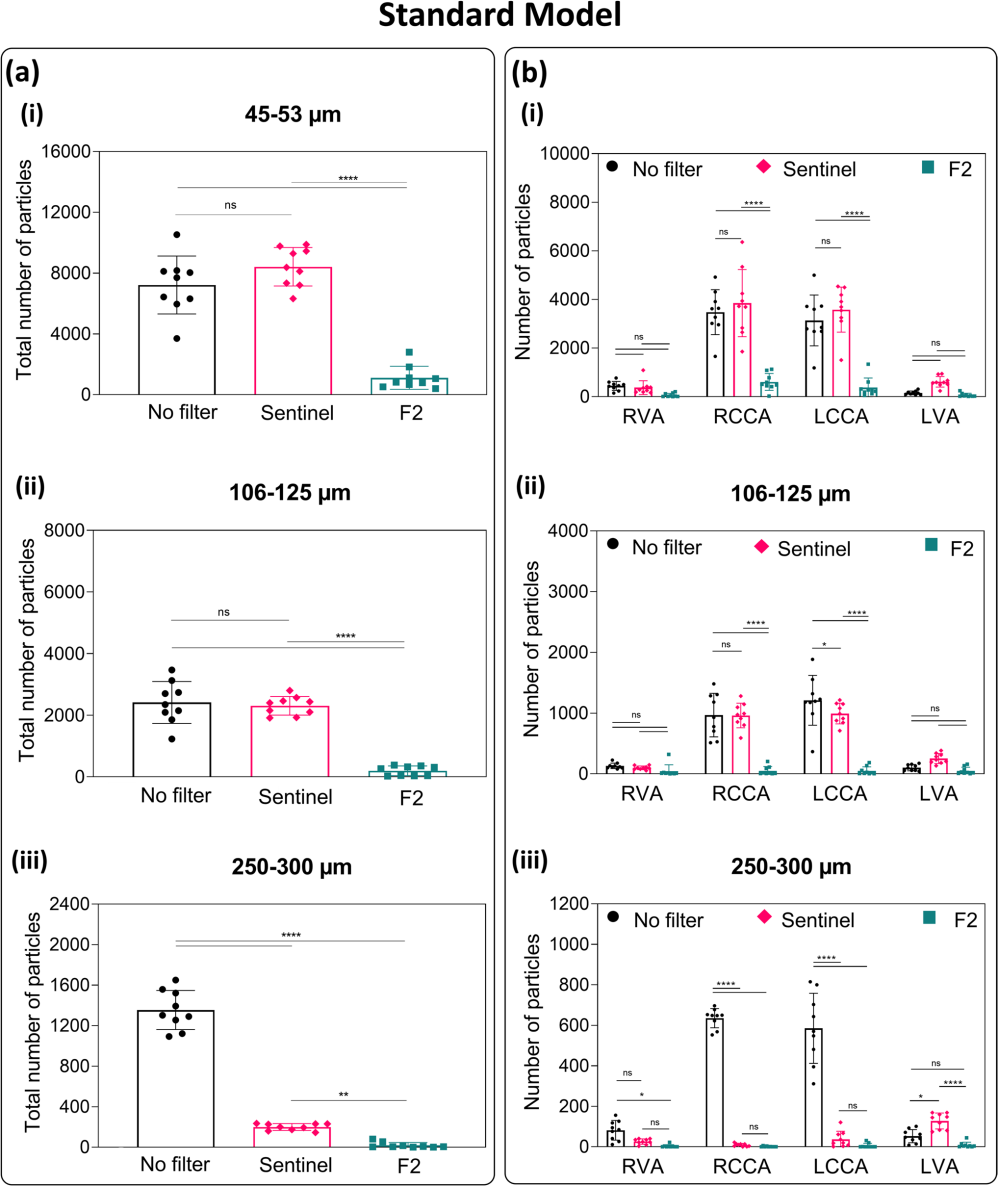
Efficacy of CEP Devices in Reducing Embolic Particles Across Cerebral Arteries in the Standard Model: **(a)** Total number of particles collected from all the four cerebral arteries with and without CEP devices, and **(b)** number of particles of variable sizes passed through cerebral arteries RVA, RCCA, LCCA, and LVA (n = 9; Three different sizes of particles were used **(i)** 45-53 µm, **(ii)** 106-125 µm, and **(iii)** 250-300 µm; asterisks mark significance levels of p ≤ 0.05 (*), p ≤ 0.01 (**), p ≤ 0.001 (***), and p ≤ 0.0001 (****)). ***Abbreviations:*** *CEP* Cerebral embolic protection, *RVA* Right vertebral artery, *RCCA* Right common carotid artery, *LCCA* Left common carotid artery, *LVA* Left vertebral artery

**Fig. 5:**
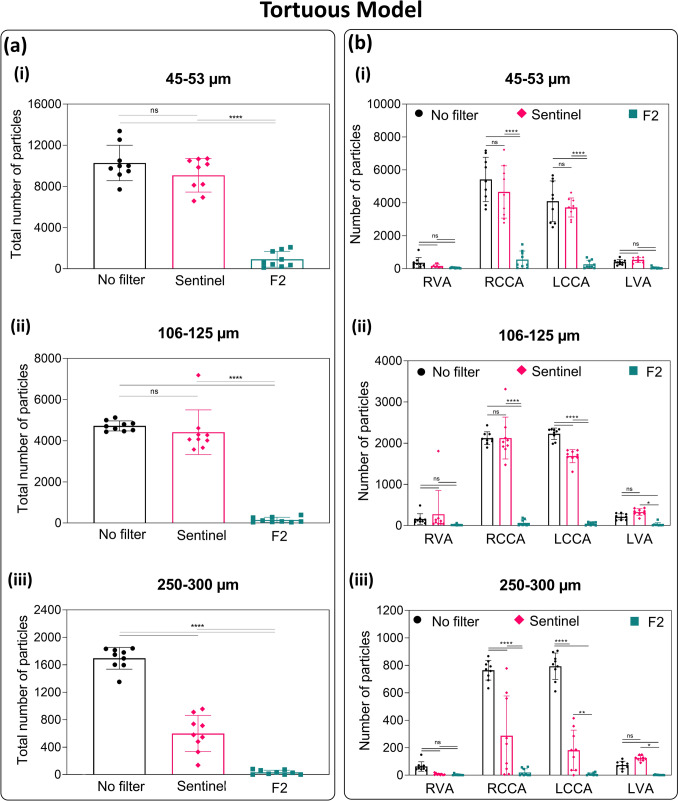
Efficacy of CEP Devices in Reducing Embolic Particles Across Cerebral Arteries in the Tortuous Model: (a) Total number of particles collected from all the four cerebral arteries with and without CEP devices, and **(b)** number of particles of variable sizes passed through cerebral arteries RVA, RCCA, LCCA, and LVA (n = 9; Three different sizes of particles were used **(i)** 45-53 µm, **(ii)** 106-125 µm, and **(iii)** 250-300 µm; asterisks mark significance levels of p ≤ 0.05 (*), p ≤ 0.01 (**), p ≤ 0.001 (***), and p ≤ 0.0001 (****)). ***Abbreviations:*** *CEP* Cerebral embolic protection, *RVA* Right vertebral artery, *RCCA* Right common carotid artery, *LCCA* Left common carotid artery, *LVA* Left vertebral artery

To evaluate the protective effect of the devices, the particles of varying sizes passing through the cerebral arteries were injected and the numbers were measured from a standard anatomical model (Figure 4) and a tortuous model (Figure 5). For visual demonstration, Videos S1, S2, and S3 showcase the injection of particles under ultraviolet light into standard silicone models under three conditions: 1) without protection, 2) with the Sentinel device, and 3) with the F2 filter for cerebral embolic protection.

Overall, the F2 filter significantly reduced particle counts across all sizes in both models compared to the control (p ≤ 0.0001). In contrast, the Sentinel device reduced only large particles (p ≤ 0.01) but showed no significant reduction for small and medium particles (Figures 4a and 5a). The protection efficacy of the Sentinel and F2 filters in the standard model was 0% and 84.6% for small particles, 4.4% and 91.8% for medium particles, and 83.3% and 99.2% for large particles, respectively. In the tortuous model, Sentinel and F2 filters demonstrated protection efficacy of 11.7% and 91.1% for small particles, 6.5% and 96.8% for medium particles, and 64.7% and 99.3% for large particles, respectively (Table 1). This result demonstrates the superior performance of the F2 filter in consistently and comprehensively protecting against embolic particles of various sizes, regardless of anatomical complexity, compared to the Sentinel device.

**Table 1:** Protection efficacy (%) of Sentinel and F2 filters in two silicone models of the human aorta (standard and tortuous models)

Particle Size (µm)	Protection Efficacy (%)			
	Standard Model		Tortuous Model	
	Sentinel	F2	Sentinel	F2
45-53	0.0	84.6	11.7	91.1
106-125	4.4	91.8	6.5	96.8
250-300	83.3	99.2	64.7	99.3

For the protection of common carotid arteries (RCCA and LCCA), the F2 filter significantly reduced the number of particles, across all particle sizes in both the standard and tortuous models (Figures 4b and 5b). However, there was no significant difference between the Sentinel device and the control group for the number of small particles that passed through the RCCA or LCCA. However, the number of medium-sized particles was reduced in the LCCA, and the number of large particles was reduced in both the RCCA and LCCA in both models.

In the RVA, although a decreasing trend in particle counts was observed with the devices, no significant differences were noted among the CEP devices and controls, except for a reduction in large particles with the F2 filter in the standard model. In the LVA, the Sentinel showed no protective effect for particles of any size, as it does not cover the left subclavian artery. In contrast, the F2 filter allowed fewer large particles to pass compared to the Sentinel in both models and fewer medium-sized particles in the standard model.

## Discussion

In this study, we investigated the effectiveness of CEP devices in protecting the cerebral circulation using standard and severe tortuous aorta models. Our results showed that the F2 device exhibited broad efficacy in protecting all cerebral arteries from particles of various sizes, without causing flow disruptions. In contrast, the Sentinel device showed limited protective efficacy, as it was unable to significantly reduce small or medium-sized particles and does not provide coverage for the left vertebral artery (LVA). The protective effect of F2 device, characterized by its small pore size (28 µm) and full anatomical coverage, indicates its promising potential to enhance cerebral protection during TAVR.

Other investigational CEP platforms include the TriGuard 3™ (Keystone Heart, a Venus Medtech company), a nitinol-frame deflector positioned in the aortic arch via an 8 Fr transfemoral sheath, offering coverage of all four cerebral branches with a ~ 115–145 µm mesh; this device is CE-marked in Europe and evaluated in the REFLECT trials [18, 22–27]. The ProtEmbo® (Protembis GmbH), by contrast, is delivered through a left radial approach and uses a 60 µm mesh designed to deflect emboli from all four brain-supplying vessels; early feasibility studies have demonstrated safety and high technical success rates [25–28], and a pivotal randomized IDE trial (PROTEMBO) is currently ongoing. However, these devices are not currently FDA-approved or commercially available in the United States. As such, the Sentinel remains the only CEP device in routine clinical use in the U.S. and was selected as the comparator for this study.

Histopathologic evaluation has shown cerebral microinfarcts as small as 50 µm detectable in the cortex and subcortex [29–32]. Diffusion-weighted MRI (DWI) also identifies acute microinfarcts up to a few millimeters [33]. Stroke rates after surgical aortic valve replacement (SAVR) are usually between 1–6% depending on the study, with most contemporary sources converging on 2–4%. DWI studies have demonstrated new ischemic lesions (silent infarcts) in 40-60% of patients after SAVR [34–36]. Multiple studies and meta-analyses support clinical stroke rates after TAVR in recent trials as about 0.6–3% in low-risk populations, with silent infarcts detected by DWI in 60–90% [37–39]. There is evidence from observational studies and meta-analyses linking silent (subclinical) ischemic lesions after SAVR and TAVR with subsequent cognitive decline, increased risk for dementia, and worsened clinical prognosis [38, 40].

Periprocedural strokes are a well-recognized cause of morbidity and mortality in patients undergoing TAVR [41, 42]. Recent trials also revealed that clinically SBIs are nearly ubiquitous among TAVR patients [12, 43, 44]. Though the clinical implications of these lesions are not yet fully understood, subclinical strokes are associated with an increased risk of cognitive decline and dementia, challenging the notion that they are entirely benign [11]. Furthermore, studies have shown that even cerebral microinfarcts (CMIs), which are classically considered undetectable on conventional MRI and only found on autopsy [29, 45], are associated with cognitive dysfunction, motor impairment, and dementia [46–51]. A recent imaging study using 3 T MRI has revealed perilesional atrophy surrounding CMIs in cortical areas much larger than the CMI core [52]. In animal studies, even a single microinfarct can impair neuronal function in regions significantly larger than the lesion core [53]. Even though most small infarctions after TAVR become undetectable on FLAIR imaging during the chronic phase, they may still contribute to long-term cognitive impairment. Therefore, reducing even small particles across all cerebral arteries is crucial in TAVR procedures.

The efficacy of the Sentinel devices has been investigated in several in randomized controlled trials, yielding variable results [5, 12, 18, 22, 54–56]. The MISTRAL-C trial reported new brain lesions on MRI in 78% of patients treated with the Sentinel device, although the lesions were numerically fewer and smaller compared to those in patients without a protection device [56]. Similarly, the CLEAN-TAVI trial showed a significant reduction in the number and size of new lesions with the Sentinel device; however, nearly all patients still had evidence of post-procedural MRI-positive lesions [10]. A multicenter trial involving 240 patients later showed no significant difference in stroke rates or new MRI lesion volume between groups [12]. More recently, a large post-market multicenter randomized controlled trial found no significant difference in periprocedural stroke within 72 hours of TAVR [57]. Notably, two of the six disabling strokes reported occurred in the posterior circulation, which is unprotected by the Sentinel device.

Our experiments are consistent with these clinical findings, showing that the Sentinel device failed to provide adequate protection for small and medium-sized particles and for the LVA. The partial protection effect with the Sentinel is due to the device’s design, which includes two filters with 140 µm pores and provides protection only to the brachiocephalic and left common carotid arteries [18, 57]. The results in this study showed that the Sentinel arm in the tortuous model exhibited lower protection efficacy for large particles than in the standard model (64.7% versus 83.3%). Outlet counts in the right CCA and the left CCA were lower than no device control yet higher than in the standard model, indicating reduced effective protection in these branches under tortuous geometry. A plausible explanation for the attenuated protection with Sentinel is geometry dependent apposition in the tortuous arteries. In the curved innominate and left CCA, the filters may not achieve complete circumferential wall contact, leaving narrow channels that permit particulate transit into these branches. These findings suggest that while the Sentinel device can reduce embolic debris, its limited anatomical coverage or ability to prevent strokes, highlight the need for comprehensive cerebral embolic protection solutions.

The benefits of the F2 design for TAVR are twofold: First, all cervical arteries are protected by its full coverage of all three great vessels. Secondly, the device offers the smallest pore size available among CEP devices. With pores spanning only 28 µm, F2 provides a distinct advantage in diverting even tiny emboli away from the cerebral circulation to preventing small emboli from causing CMI. This in vitro study demonstrated the superior efficacy of the F2 filter in preventing particles of all sizes (40-300 µm) from entering the cerebral circulation compared to both the Sentinel device and unprotected controls (Figures 4 & 5). This superior efficacy was achieved without compromising blood flow through the great vessels (Figures 3). Additionally, the protection effect with F2 filter was not affected by the tortuous anatomy, whereas Sentinel was less effective in these conditions, with a higher number of particles observed when used in the tortuous model compared to the standard model. This is consistent with prior studies which have shown that complex anatomy of the aortic arch and supra-aortic vessels can be challenging with Sentinel [57–59].

Notably, the F2 filter was recently used successfully in a first-in-human study involving three TAVR procedures, without any complications [60]. This initial success is promising but still requires further validation in larger cohorts.

A limitation of the study is the use of in vitro models and the use of synthetic particles, which does not fully replicate the complexity of real-world clinical scenarios. However, these models offer several advantages. Realistic aortic models with physiologically relevant flow dynamics closely mimic human anatomy, ensuring the clinical relevance of the findings. Additionally, in vitro studies allow for multiple replicates, an aspect that is challenging to achieve in clinical studies due to patient variability and ethical considerations. In clinical settings, it is impossible to accurately quantify or control the number of embolic debris during TAVR. Furthermore, direct comparisons of different devices in patients under identical conditions are not feasible, making in vitro models a valuable alternative for controlled and reproducible evaluations. A further limitation is the use of a peristaltic pump, which lacks the physiologic pulsatile waveform that may influence embolus trajectories. Although the peristaltic pump generates a quasi-pulsatile waveform and we applied realistic mean flow volume in each branch across conditions, it does not reproduce the physiologic arterial pattern, potentially biasing estimates of device capture/deflection. Importantly, we matched mean total flow and branch distributions across all conditions, so the observed between-device differences are most plausibly attributable to device design including pore size and anatomic coverage rather than the pulsatility. Nevertheless, the results may be biased by the flow condition. Additionally, we did not record inlet or outlet pressures, so we cannot report device related pressure drop. However, measured flows in the four cerebral branches did not decrease with the F2 device compared with the no device control, indicating that cerebral perfusion was preserved under our test conditions.

## Conclusion

This study demonstrates the protection effect of the F2 filter in the in vitro experimental models. Future randomized controlled trials are needed to establish the efficacy of the F2 filter in preventing cerebral embolism during TAVR and neurocognitive benefits.

## Supplementary Information

Below is the link to the electronic supplementary material.

Supplementary file1 (MP4 16639 KB)

Supplementary file2 (MP4 17262 KB)

Supplementary file3 (MP4 9631 KB)

## Data Availability

The data that support the findings of this study are available on request from the corresponding author.
